# Linking mechanistic and behavioral responses to sublethal esfenvalerate exposure in the endangered delta smelt; *Hypomesus transpacificus *(Fam. Osmeridae)

**DOI:** 10.1186/1471-2164-10-608

**Published:** 2009-12-15

**Authors:** Richard E Connon, Juergen Geist, Janice Pfeiff, Alexander V Loguinov, Leandro S D'Abronzo, Henri Wintz, Christopher D Vulpe, Inge Werner

**Affiliations:** 1 School of Veterinary Medicine, Department of Anatomy, Physiology and Cell Biology, University of California, Davis, California 95616, USA; 2 School of Veterinary Medicine, Molecular Biosciences, University of California, Davis, California 95616, USA; 3 School of Nutritional Sciences and Toxicology, University of California, Berkeley, California 94720, USA; 4 Unit of Functional Aquatic Ecology and Fish Biology, Department of Animal Science, Technische Universität München, D-85350 Freising, Germany; 5 Biorad Laboratories, Life Science Research, Hercules, California, USA

## Abstract

**Background:**

The delta smelt (*Hypomesus transpacificus*) is a pelagic fish species listed as endangered under both the USA Federal and Californian State Endangered Species Acts and considered an indicator of ecosystem health in its habitat range, which is limited to the Sacramento-San Joaquin estuary in California, USA. Anthropogenic contaminants are one of multiple stressors affecting this system, and among them, current-use insecticides are of major concern. Interrogative tools are required to successfully monitor effects of contaminants on the delta smelt, and to research potential causes of population decline in this species. We have created a microarray to investigate genome-wide effects of potentially causative stressors, and applied this tool to assess effects of the pyrethroid insecticide esfenvalerate on larval delta smelt. Selected genes were further investigated as molecular biomarkers using quantitative PCR analyses.

**Results:**

Exposure to esfenvalerate affected swimming behavior of larval delta smelt at concentrations as low as 0.0625 μg.L^-1^, and significant differences in expression were measured in genes involved in neuromuscular activity. Alterations in the expression of genes associated with immune responses, along with apoptosis, redox, osmotic stress, detoxification, and growth and development appear to have been invoked by esfenvalerate exposure. Swimming impairment correlated significantly with expression of aspartoacylase (ASPA), an enzyme involved in brain cell function and associated with numerous human diseases. Selected genes were investigated for their use as molecular biomarkers, and strong links were determined between measured downregulation in ASPA and observed behavioral responses in fish exposed to environmentally relevant pyrethroid concentrations.

**Conclusions:**

The results of this study show that microarray technology is a useful approach in screening for, and generation of molecular biomarkers in endangered, non-model organisms, identifying specific genes that can be directly linked with sublethal toxicological endpoints; such as changes in expression levels of neuromuscular genes resulting in measurable swimming impairments. The developed microarrays were successfully applied on larval fish exposed to esfenvalerate, a known contaminant of the Sacramento-San Joaquin estuary, and has permitted the identification of specific biomarkers which could provide insight into the factors contributing to delta smelt population decline.

## Background

The delta smelt (*Hypomesus transpacificus*) is a pelagic fish species endemic to the Sacramento-San Joaquin estuary, whose abundance has dramatically declined since the 1980s, and more precipitously in recent years [1-4]. It was listed as endangered in 2009, under both the Federal Endangered Species Act (ESA) and California Endangered Species Act (CESA). Considerable efforts are presently being made to understand the causes of this recent population decline [4,5], especially because several other pelagic species have shown similar population trends. Delta habitats have been compromised by a number of complex factors, both known and unknown, potentially affecting aquatic species throughout the Sacramento-San Joaquin watersheds and estuary [4]. Pollution, in the form of chemicals contained in runoff from agricultural and urban areas, and old mining sites, treated wastewater effluent, along with the effects of water exports, invasive species and habitat destruction are amongst potential causes for the population decline of several pelagic species [5].

Identifying the sublethal impacts of environmental stressors and their mechanistic effects on resident individuals and populations is a major challenge in ecotoxicology. Contaminants may not only affect organism survival, but can compromise ecological fitness of individual species via sublethal physiological, behavioral or immunological effects (e.g. [6-10]), consequently altering food web and ecosystem dynamics. However, such physiological endpoints are often difficult to determine in field studies, because they either require behavioral observation and measurements, or because affected individuals will not survive in the wild. Similarly, widely used ecotoxicological tools such as standard toxicity tests [11,12] cannot easily be adapted to resident species of concern, and, conversely, it is problematic to extrapolate test results obtained with surrogate species to resident species of concern [13]. Recent comparative studies have demonstrated a need for identifying effects directly in the species of concern, as traditional model organisms may differ in sensitivity and physiological response to environmental contaminants and other stressors [14,15].

Carefully selected molecular biomarkers can provide species-specific and sensitive, mechanistic information on the overall health of an organism, as toxic responses are often preceded by alterations in gene expression [16,17]. In particular microarray gene profiling is a powerful tool for defining genome-wide effects of environmental change on biological function [16,18,19]. The predictive value of microarrays as screening tools, as well as our understanding of these responses and their application in the field of ecotoxicology is rapidly growing. This technology can be applied in vertebrates and invertebrates, plants, algae, cell lines and unicellular organisms [20]. In addition, links are being established between specific molecular biomarkers identified by microarray technology, and higher-level life history parameters, such as metabolism, growth and reproduction [16,18,21,22]. Gene expression studies carried out over short-term exposures have allowed for the prediction of chronic stressor effects, such as reduced fecundity and embryonic arrest, somatic growth, and population dynamics [16,18,21,23]. Thus, specific gene responses in studied organisms would not only be indicative of health status, but when used in conservation studies, could highlight potential causes for population decline. However, few biomarkers are currently understood well enough to provide conclusive evidence of contaminant impacts on aquatic species in field monitoring, and extrapolating effects seen at the biomarker level to individual or population-level toxicity continues to be a challenge. For molecular biomarkers to be used as successful monitoring tools of individual, population and ecosystem damage, strong links need to continue to be established between gene expression and health status.

To better understand the sublethal effects of contaminants upon *H. transpacificus*, and to identify biomarkers for future field investigations, we have constructed a microarray with 8,448 Expressed Sequence Tags (ESTs). No genomic information was available on any database at the time this project began, other than a few mitochondrial sequences used in taxonomic studies [24]. We describe here, the construction and first application of this tool to identify genes in the delta smelt, specifically responding to exposure to esfenvalerate, a pyrethroid insecticide, and present gene expression quantitation of selected biomarkers, utilizing these to explain observed swimming abnormalities. We used esfenvalerate in our study because biochemical responses and adverse effects on the whole organisms are relatively well understood [25] and therefore would aid interpretation of results in this "proof of principle" test. Esfenvalerate [(*S*)-a-cyano-3-phenoxybenzyl-(*S*)-2-(-4-chlorophenyl)-3-methylbutyrate] is a synthetic pyrethroid insecticide, widely used in agriculture, with a high risk to aquatic organisms [26]. It causes neurological damage by blocking sodium and potassium channels, resulting in repetitive neurological discharge [25]. In addition, pyrethroid insecticides are highly soluble in myelin sheaths of nerves, causing demyelination, resulting in conduction deficiencies through nerve lesions [27], directly affecting swimming ability, and impinging on foraging and migration. Fish are highly sensitive to this insecticide, with for example effects on bluegill behavior at measured concentrations as low as 0.025 μg/L^-1^[28]. Pyrethroids have also been reported to affect growth, induce immune responses, reduce hepatic glycogen levels and delay spawning [9,29].

The main focus of this study was not the development of the microarray, rather the identification of molecular biomarkers specific to the delta smelt and stressors found in the San Joaquin-Sacramento delta. We present here results from annotated genes identified through microarray analyses and specifically quantitative PCR analyses of selected molecular biomarkers.

## Results and Discussion

### Effects of esfenvalerate exposure: mortality and swimming behavior

Fish larvae are known to be highly sensitive to esfenvalerate, with effects on swimming performance and enhanced susceptibility to predation resulting from concentrations as low as 0.0625 μg/L^-1^[10]. Behavior alterations are construed as being consequential to the reported neurological mode of action of this pesticide, further affecting foraging, migration and reproduction [30]. Toxicity of pyrethroids in the Sacramento-San Joaquin estuary is likely alleviated by the presence of particles and organic matter, and to date concentrations of esfenvalerate detected in the water column were low, however, concentrations in winter storm runoff from agricultural lands have been reported up to 0.093 μg.L^-1 ^[31], influencing our decision to investigate dose response exposures to both high and environmentally relevant concentrations in confirmatory studies.

**Figure 1:**
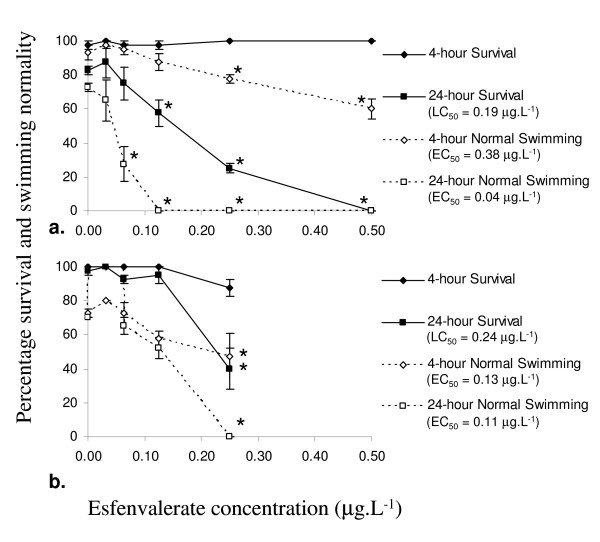
**Swimming behavior and mortality**. Percentage swimming normality and survival in (a.) 10-d old and (b.) 52-d old *H. transpacificus *exposed to esfenvalerate, ± standard errors (n = 10). * Indicates significant reduction in survival or swimming performance compared to solvent control.

In terms of mortality, 10-d old delta smelt were only slightly more sensitive in this study (LC_50,24 h _= 0.19 μg.L^-1^) than 52-d old (LC_50,24 h _= 0.24 μg.L^-1^), however swimming performance of the younger larvae was affected at a concentration approximating one third of that observed affecting older fish (figure 1). Swimming abnormality in 10-d old larvae, intensified with increasing esfenvalerate concentration at 4 h, escalating significantly after 24 h exposure (figure 1a). This swimming abnormality was also concentration dependent in 52-d old fish, however swimming effects resulting from different time point measurements differed only at the highest exposure concentration of 0.250 μg.L^-1 ^(figure 1b), where effects on motion increased from 22.5% anomaly at 4 h to 45% at 24 h. Behavioral abnormalities, reduced food intake and growth, as well as increased susceptibility to predation were reported in fathead minnow larvae exposed to esfenvalerate for 4 h to concentrations above 0.455 μg.L^-1 ^[10]. Significant swimming impairments were determined in this study at 0.250 μg.L^-1^, thus delta smelt are highly sensitive to sublethal esfenvalerate exposure. Furthermore, bioaccumulation in rainbow trout have resulted in concentrations 400 times higher than background ambient levels http://extoxnet.orst.edu.

### Microarray application and q-PCR

**Figure 2:**
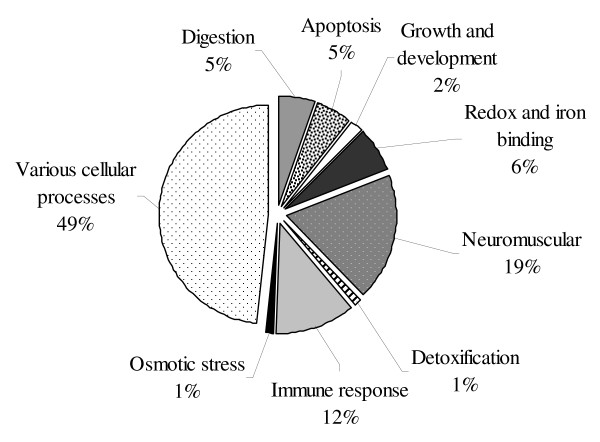
**Functional classification of gene expression**. Functional classification of genes responding to esfenvalerate exposure (0.0625 and 0.125 μg.L^-1^) in 52-d old delta smelt. Percentages were calculated based on function and biological processes of 94 unique differentially expressed genes.

Through the application of the delta smelt microarrays, and combined analytical methods, we have identified 288 ESTs, from which a number of genes of interest could be used to measure the effect of esfenvalerate and potentially other pyrethroid insecticides; further investigating their use as biomarkers in this species. Of the sequenced ESTs that responded significantly, 118 genes were successfully annotated; 170 matched unnamed or hypothetical proteins, or did not match the described annotation selection criteria; i.e. BLASTx searches resulted in expect-values greater than 1 × 10^-5 ^and scores below 50. Based on gene ontology; molecular function, biological processes and cellular components, 94 unique genes were functionally classified (figure 2 and table 1) and described below. Based on the proportions of ESTs responding to successfully annotated unique genes (33%), it is estimated that of the 8,448 ESTs printed on the microarray, there could be above 2,500 unique genes identified in the delta smelt. These numbers however represent responses to a single contaminant, and should not be construed as final as there will have an intrinsic bias exerted upon them, however, the proportion of repeated sequences in the analyses was very low, with a maximum of nine repetitions for CHK1 checkpoint homologue and not more than one or two duplication for a few others. It is also important to note that the microarray was manufactured with incomplete genome data, thus information presented in figure 2 represents proportions of a limited number of available genes.

**Table 1:** Classification of differential gene expression of esfenvalerate responding genes in 52-d old *H. transpacificus *following 24 h exposure as identified by microarray analyses.

BLASTX top hit	Species match	Accession No.	Score	E-value	Concentration	Fold Change (+/-)	P-VALUE (or cut-off)	
Neuromuscular								
**titin a**	*Danio rerio*	ABG48500	110	4.00E-23	0.0625	1.3277	0.0625	**
smooth muscle cell-specific protein SM22 alpha	*Epinephelus coioides*	ABW04145	349	1.00E-94	0.1250	1.2252	0.0029	
toxin-1	*Oncorhynchus mykiss*	AAM21198	116	5.00E-25	0.1250	1.1884	0.0044	
thymosin beta-12	*Lateolabrax japonicus*	P33248	80	2.00E-13	0.1250	1.1876	0.0003	
similar to 19.9 kD myosin light chain isoform 1	*Danio rerio*	XP_685183	332	1.00E-89	0.1250	1.1720	0.0046	
ictacalcin	*Ictalurus punctatus*	AAY86967	145	1.00E-33	0.1250	1.1607	0.0021	
tropomyosin	*Theragra chalcogramma*	BAC44994	281	2.00E-74	0.1250	1.1368	0.0090	
N-acylsphingosine amidohydrolase	*Takifugu rubripes*	AAM43813	367	e-100	0.1250	1.1196	0.0082	
alanine-glyoxylate aminotransferase	*Platichthys flesus*	CAH59400	345	2.00E-93	0.1250	1.0860	0.0059	
titin b	*Danio rerio*	ABG48499	65	5.00E-09	0.1250	-1.0841	0.0045	
alpha-2,8-polysialyltransferase IV	*Oncorhynchus mykiss*	BAC77411	70	1.00E-10	0.1250	-1.0878	0.0030	
hedgehog acyltransferase-like, a	*Danio rerio*	NP_957181	295	1.00E-78	0.1250	-1.1419	0.0074	
**parvalbumin**	*Cyprinus carpio*	CAC83659	173	7.00E-42	0.1250	-1.1675	0.0033	
BTEB transcription factor	*Pimephales promelas*	ABO28528	107	1.00E-21	0.0625	-1.2019	0.0625	*
myosin regulatory light chain 2	*Salmo salar*	CAD89610	330	7.00E-89	0.1250	-1.2121	0.0020	
similar to Clca1 protein	*Danio rerio*	XP_694323	198	2.00E-49	0.0625	-1.2547	0.0625	*
ependymin	*Perca flavescens*	ABU49423	168	2.00E-40	0.0625	-1.2602	0.0625	*
**aspartoacylase**	*Danio rerio*	NP_001103573	384	e-105	0.0625	-1.3905	0.0625	*
Immune								
carboxypeptidase B	*Paralichthys olivaceus*	BAC53789	365	2.00E-99	0.1250	1.2897	0.0041	
fish-egg lectin (FEL)	*Cyprinus carpio*	P68512	192	2.00E-47	0.1250	1.1769	0.0003	
procathepsin B	*Oncorhynchus mykiss*	AAK69705	346	1.00E-93	0.0625	-1.1727	0.0625	*
gamma-glutamyl hydrolase	*Danio rerio*	NP_998487	223	6.00E-57	0.0625	-1.1939	0.0625	*
membrane glycoprotein	*Human coronavirus*	ABD75532	53	1.00E-05	0.0625	-1.1945	0.0625	*
**beta-2 microglobulin**	*Salmo salar*	AAG17525	176	8.00E-43	0.1250	-1.1995	0.0034	
microtubule-associated protein 1 light chain 3 alpha	*Danio rerio*	NP_999904	238	3.00E-61	0.0625	-1.2245	0.0625	*
microtubule-associated protein, RP/EB family, member	*Danio rerio*	NP_998805	272	1.00E-71	0.0625	-1.2298	0.0625	*
T-cell receptor beta chain ANA 11, putative	*Brugia malayi*	EDP38115	63	2.00E-08	0.1250	-1.2600	0.0052	
glycerophosphodiester phosphodiesterase domain containing 1	*Danio rerio*	NP_001004118	322	2E-86	0.0625	-1.2652	0.0625	*
CHK1 checkpoint homolog	*Xenopus tropicalis*	CAJ83813	92	2.00E-17	0.1250	-1.3981	0.0013	
Apoptosis								
tissue inhibitor of metalloproteinase 2	*Oncorhynchus mykiss*	AAU14867	265	3.00E-69	0.1250	1.1235	0.0082	
cathepsin H	*Danio rerio*	NP_997853	300	5.00E-80	0.1250	1.0898	0.0069	
**caspase-3**	*Dicentrarchus labrax*	ABC70996	223	6.00E-68	0.1250	-1.0647	0.0022	
caspase-1	*Dicentrarchus labrax*	ABB05054	79	3.00E-13	0.1250	-1.1270	0.0061	
cathepsin S-like	*Oncorhynchus mykiss*	AAV32964	291	1.00E-77	0.1250	-1.1507	0.0047	
Redox and metal ion binding								
hydroxymethylbilane synthase	*Danio rerio*	CAM15096	369	e-101	0.1250	1.3333	0.0042	
**hemopexin**	*Danio rerio*	NP_001104617	313	2.00E-83	0.1250	1.2968	0.0052	
transferrin	*Salvelinus fontinalis*	BAA84100	326	1.00E-87	0.1250	1.1769	0.0080	
similar to leprecan-like 1 protein	*Danio rerio*	XP_695073	183	1.00E-44	0.1250	1.1543	0.0036	
similar to synaptic glycoprotein SC2	*Danio rerio*	XP_693420	430	e-119	0.1250	-1.1340	0.0073	
similar to LOC407663 protein	*Danio rerio*	XP_698537	124	6.00E-27	0.0625	-1.2429	0.0625	*
Growth and development								
yghl1 (Putative growth hormone like protein-1)	*Seriola quinqueradiata*	BAB62526	153	1.00E-35	0.1250	-1.1388	0.0072	
**ZPA domain containing protein**	*Oryzias latipes*	NP_001098216	168	3.00E-40	0.0625	-1.1568	0.0625	*
Detoxification								
**pregnane × receptor**	*Oncorhynchus mykiss*	ABP38412	206	2.00E-51	0.0625	-1.2321	0.0625	*
Osmotic stress								
hyperosmotic glycine rich protein	*Salmo salar*	AAO32675	134	8.00E-30	0.1250	-1.1053	0.0021	
Digestion								
similar to Apoa4 protein isoform 2 (Apolipoprotein)	*Danio rerio*	XP_698920	296	7.00E-79	0.1250	1.4959	0.0056	
chymotrypsinogen 2-like protein	*Sparus aurata*	AAT45254	460	e-128	0.1250	1.4211	0.0095	
pancreatic carboxypeptidase A1 precursor copy 2	*Tetraodon nigroviridis*	AAR16321	242	9.00E-63	0.1250	1.3204	0.0025	
pancreatic protein with two somatomedin B domains	*Paralichthys olivaceus*	BAA88246	214	e-100	0.1250	1.1297	0.0021	
chitinase (Zgc:55941)	*Danio rerio*	AAH44549	369	e-100	0.1250	-1.0618	0.0097	

Genes selected for quantitative PCR analyses are shown in bold. * indicates genes responding at 0.0625 μg.L^-1 ^esfenvalerate and ** indicates gene responding to both 0.0625 and 0.125 μg.L^1 ^esfenvalerate. Remaining genes responded only at 0.125 μg.L^1 ^esfenvalerate exposure. Information represents proportions of a limited number of available genes on the applied microarray.

Differences between methods used allow for greater mining of possible biomarkers. The method by Loguinov *et al *[32] identified a single differentially expressed gene at 0.125 μg/L^-1^ esfenvalerate (with no significant homology). The addition of LIMMA GUI analysis, also identifying this single gene, generated a broader list of genes for biomarker selection.

A large percentage of uniquely annotated genes, responding to esfenvalerate exposure; 49%, were classified as involved in various biological processes. These included genes encoding for ribosomal proteins, t-RNA synthases, telomerases, uncoupling proteins and genes involved in chromosome maintenance. Of greater interest was the identification of genes involved in neuromuscular activity; representing 19% of identified sequences, a further 12% eliciting immune responses, along with 6% related to oxidative stress, respiration and iron storage and 5% relevant to apoptosis. Digestion appears to have also been affected, along with growth and development, represented by genes coding for pancreatic enzymes, a zona pellucida protein; inferred to be choriogenin, and a growth hormone.

A selected group of genes, highlighted in bold in the gene list in table 1, were validated using q-PCR and further investigated for use as molecular biomarkers. These results are described below, in conjunction with differentially expressed genes identified through microarray application.

### Specific effects of esfenvalerate exposure on gene expression

**Figure 3:**
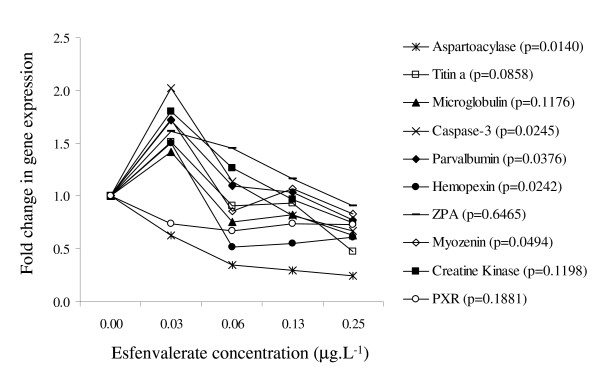
**Molecular biomarkers responses**. Fold changes in gene expression of ten selected biomarkers in esfenvalerate exposed, 10-d old, delta smelt, assessed by quantitative PCR. Significance in expression differences, as determined by One-way ANOVA, is shown in brackets in legend.

Pyrethroid insecticides are known sodium and potassium channel modulators [25], with axon demyelinating effects [27]. This the proof-of-principle microarray assessment of esfenvalerate exposure of 52-d old delta smelt, was successfully used to screen for, and further understand its mode of action, identifying neuromuscular responses, that were confirmed as highly significant through qPCR, corroborating known effects of this pesticide, but also pointing at other significant effects upon growth and development, digestion and the immune system. Gene expression assessed by qPCR on 10-d old delta smelt that verified these genomic responses are presented below. Correlations in expression between q-PCR investigated genes are shown in table 2 and fold changes in gene expression are summarized in figure 3 and table 3.

**Table 2:** Pairwise correlations of gene expression in esfenvalerate exposed 10-d old delta smelt.

	Aspartoacylase	Titin a	Microglobulin	Caspase-3	Parvalbumin	Hemopexin	ZPA	Myozenin	Creatine Kinase	PXR
Aspartoacylase	1 (0)									
Titin a	0.490 (0.402)	1 (0)								
Microglobulin	0.576 (0.310)	0.954 (**0.012**)	1 (0)							
Caspase-3	0.344 (0.571)	0.919 (**0.027**)	0.921 (**0.026**)	1 (0)						
Parvalbumin	0.284 (0.644)	0.953 (**0.012**)	0.928 (**0.023**)	0.981 (**0.003**)	1 (0)					
Hemopexin	0.629 (0.256)	0.819 (0.090)	0.951 (0.013)	0.860 (0.061)	0.822 (0.088)	1 (0)				
ZPA	-0.017 (0.979)	0.788 (0.113)	0.646 (0.239)	0.853 (0.066)	0.871 (0.054)	0.472 (0.422)	1 (0)			
Myozenin	0.309 (0.613)	0.898 (**0.038**)	0.949 (**0.014**)	0.909 (**0.033**)	0.943 (**0.016**)	0.892 (**0.042**)	0.677 (0.210)	1 (0)		
Creatine Kinase	0.255 (0.679)	0.916 (**0.029**)	0.865 (0.058)	0.984 (**0.003**)	0.978 (**0.004**)	0.759 (0.137)	0.932 (**0.021**)	0.859 (0.062)	1 (0)	
PXR	0.880 **(0.049)**	0.087 (0.890)	0.208 (0.737)	-0.113 (0.857)	-0.153 (0.805)	0.307 (0.616)	-0.475 (0.418)	-0.038 (0.952)	-0.218 (0.725)	1 (0)

Numbers represent correlation coefficients; r, and significance probabilities; (p), between ten selected biomarkers assessed with quantitative PCR. Bold p-values highlight significant correlations.

**Table 3:** Mean and standard deviations in fold changes in gene expression of ten selected biomarkers in esfenvalerate exposed, 10-d old, delta smelt, assessed by quantitative PCR.

Gene\Concentration		0.000	0.031	0.063	0.125	0.250
Aspartoacylase	Mean	1.000	0.629	0.346	0.299	0.244
	*SE*	*0.240*	*0.100*	*0.078*	*0.035*	*0.088*
Titin	Mean	1.000	1.515	0.909	0.933	0.475
	*SE*	*0.529*	*0.198*	*0.095*	*0.192*	*0.096*
Microglobulin	Mean	1.000	1.420	0.760	0.828	0.628
	*SE*	*0.404*	*0.127*	*0.104*	*0.154*	*0.221*
Caspase	Mean	1.000	2.024	1.136	0.818	0.670
	*SE*	*0.336*	*0.432*	*0.043*	*0.117*	*0.094*
Parvalbumin	Mean	1.000	1.718	1.097	1.037	0.771
	*SE*	*0.241*	*0.151*	*0.125*	*0.062*	*0.107*
Hemopexin	Mean	1.000	1.501	0.521	0.548	0.612
	*SE*	*0.089*	*0.296*	*0.051*	*0.147*	*0.295*
ZPA	Mean	1.000	1.612	1.455	1.165	0.912
	*SE*	*0.415*	*0.321*	*0.224*	*0.210*	*0.574*
Myozenin	Mean	1.000	1.730	0.857	1.069	0.835
	*SE*	*0.212*	*0.205*	*0.121*	*0.093*	*0.188*
Creatine Kinase	Mean	1.000	1.799	1.265	0.968	0.750
	*SE*	*0.270*	*0.249*	*0.272*	*0.162*	*0.145*
PXR	Mean	1.000	0.737	0.668	0.737	0.729
	*SE*	*0.126*	*0.083*	*0.124*	*0.080*	*0.178*

#### i. Neuromuscular responses

Parvalbumin expression in 10 d old larvae was induced 1.8-fold (t-test, p = 0.008) at 0.0313 μg.L^-1 ^esfenvalerate, reducing in expression at higher concentrations. Localized in fast-contracting muscles, some endocrine tissues, in the nervous system in GABAergic interneurons and in the brain [33], parvalbumin removes calcium from myofibrils, protecting neurons from hyper-excitability and facilitating muscle relaxation [34]. Accumulation of calcium in muscular tissue contributes to muscle degradation, muscular dystrophy and muscle fiber necrosis [35]. Estrogen is required for parvalbumin expression, thus estrogen receptor-β co-expresses with parvalbumin [36]. Estrogen is also required in brain development and has a protective neurological role, by regulating the activity of GABAergic systems within the hippocampus, basal forebrain and hypothalamus [37]. Differential expression of parvalbumin on exposure to esfenvalerate may be resultant of estrogenic effects. Pyrethroid pesticides have steroid receptor-binding activity [38] linked with endocrine disruption [39], thus exposure is likely to affect the population dynamics of wildlife not only through neuromuscular impairments, but also by affecting reproductive output [29,40]. Parvalbumin, could therefore, be a good indicator of possible endocrine-disruption as well as neuromuscular impairments.

Interestingly, expression of aspartoacylase (ASPA) in exposed 10-d old delta smelt larvae was significantly affected at all concentrations, downregulating with increasing esfenvalerate concentration in a dose response manner, and correlating significantly with swimming anomaly at 24 h (r = 0.913, p = 0.029). Aspartoacylase catalyzes hydrolysis of *N*-acetyl-L-aspartate (NAA) to aspartate and acetate in the vertebrate brain [41]. Variations in NAA measured in urine, blood and brain, have been used as diagnosis of nervous system diseases such as Alzheimer's and multiple sclerosis [42,43]. Measurements of NAA, along with ADP levels determined by creatine kinase activity, are used to evaluate the energetic state of the brain, a positive linear correlation existing between NAA and ADP synthesis [44]. Deficiency in ASPA activity leads to degeneration of the myelin; an ensheathment that isolates and controls axonal activity, it is associated with schizophrenia [45], and is the established cause of leukodystrophy in Canavan's disease [43]. Abnormal myelination is known to result from acyltransferase deficiency [46]. Ependymin, a myelin associated glycoprotein related to memory formation and involved in neuronal regeneration [47], was also negatively affected by esfenvalerate. Myelin has been postulated as a probable modulator of ASPA activity [48] further affecting this critical pathway of neurological function. ASPA protein activity is a strong biomarker of brain damage and neurological impairment investigation, used regularly in human and veterinary disease diagnostics [49].

Creatine kinase was significantly up-regulated at 0.0313 μg.L^-1 ^esfenvalerate (t-test, p < 0.05). Creatine kinase protein is used not only as a diagnosis of brain energetic value as mentioned above, but also of diseases like cardiac infarction and skeletal muscle necrosis [50]. In muscle, creatine kinase is specifically bound to sarcoendoplasmic reticulum, and regulates calcium uptake and ATP/ADP ratios [51], thus is directly involved in muscle contraction. Of interest here, are the pathway links and correlating responses (r = 0.98) between parvalbumin; facilitating muscle relaxation by binding calcium, and creatine kinase, which regulates calcium uptake. These two parameters on their own indicate muscular activity impairments, creating strong links with observed larval swimming behavior.

Titin expression also correlated significantly with parvalbumin (r = 0.95) and creatine kinase (r = 0.92), though not statistically significant in q-PCR assessments of 10-d old smelt larvae. Titin is an important protein also involved in muscle contraction, responsible for muscle elasticity and is the molecular scaffold for thick actin filament formation, forming a connection between filaments and the muscle Z-line [52]. Myozenin, another protein involved in muscle contraction, was significantly influenced by esfenvalerate exposure (t-test, p < 0.05) in 10-d old larvae. Co-regulating with Titin (r = 0.90), myozenin is a Z-line, α-actinin- and γ-filamin-binding protein expressed predominantly in skeletal muscle, and has been suggested as a biomarker for muscular dystrophy and other neuromuscular disorders [53].

#### ii Immune responses

In this study we have identified a significant alteration in expression of several genes involved in immune responses, most of them with links to neurological damage. β-microglobulin, a small protein normally found on the surface of many cells, including lymphocytes, is known to be involved in cell protection [54]. β-microglobulin is almost exclusively catabolized in the kidney and its excretion is an indication of long term nephrotoxicity [55]. High concentrations of β-microglobulin are reported to inhibit generation of functional dendritic cells [56], thus an increased amount in the blood or urine may be a sign of neural degeneration and of certain diseases, including some types of cancer, such as multiple myeloma or lymphoma. β-microglobulin levels are also reported to rise following viral infection and its reduced expression can compromise the immune system [57]. Interestingly, exposure to esfenvalerate resulted in a significant increase of pathogen susceptibility in chinook salmon [9]. β-microglobulin assessment with q-PCR did not show any significance in expression in 10 d old larvae (t-test, p = 0.131), however, an overall increase was observed at low concentrations of esfenvalerate, correlating significantly with expression of other genes investigated, further discussed below.

Multiple sclerosis is caused by an immunological attack on myelin [58], decreasing NAA and resulting in neurological instability. Furthermore, oxidative stress is inductive of apoptosis of myelin-reactive T cells [59]. A putative T cell receptor gene was identified through microarray screening, probably reacting to pyrethroid exposure, acting upon the myelin sheath and causing further neurological damage and cell death.

#### iii Apoptotic responses

Caspases (*c*ysteine-*asp*artic acid prote*ase*) belong to a family of cysteine proteases that cleave other proteins, such as the precursor forms of the inflammatory cytokines interleukin 1-β and interleukin 18, into active mature peptides and are also involved in programmed cell death; or apoptosis [60]. Enzymatic activity requires an aspartic acid residue, and plays a critical role in the regulation of proinflammatory cytokines [61] that are associated with septic shock and autoimmune syndromes [62]. Upregulation of proinflammatory cytokines were reported in viral infected salmon, which further increased in expression following esfenvalerate exposure [63]. Caspases contribute to the pathogenesis of neurodegenerative disorders such as ischemia, Krabbes and Huntington's diseases, Alzheimer's, and other leukodystrophic diseases resulting in neural degeneration [64,65]. Moreover, caspase inhibitors have been suggested as therapeutic treatments for neurodegenerative diseases [66]. Low concentrations of esfenvalerate; 0.0313 μg.L^-1^, significantly induced caspase-3 expression 2-fold (t-test, p = 0.002) in 10-d old delta smelt. As caspases are activated by aspartic acid, induction may be suggestive of increases in substrate residues, along with inflammatory cytokines, probable effects upon the immune system, and subsequent neurodegeneration. Furthermore, a decrease in ASPA expression could be suggestive of reduced breakdown of NNA to aspartate and acetate, required as substrate for caspase activity, and synthesis of proteins required in repair mechanisms.

#### iv. Redox and metal ion binding

Upregulation of hemopexin was confirmed by quantitative PCR in 10-d old delta smelt exposed to 0.0313 μg.L^-1 ^esfenvalerate. Hemopexin-like protein, a gene sequence displaying vast similarities to warm-temperature-acclimation-related-65 protein (WAP65) on BLAST homologies with Japanese medaka (*Oryzias latipes*), was identified as significantly upregulated through microarray screening. Hemopexin is synthesized by Schwann cells following nerve injury [67], accumulation has been reported in the peripheral nervous system following axonal lesions, and is specifically regulated during repair, returning to normal levels on axonal regeneration [68]. Wallerian degeneration occurs after axonal injury and is critical for repair, it is characterized by axonal and myelin degeneration [69], is accompanied by macrophage invasion and subsequent synthesis of hemopexin [67]. Hemopexin appears to play a significant role in neural regeneration, but may be resultant of oxidative stress mediated T cell activity on myelin sheaths (described above, under immune responses). Though upregulation follows nerve injury and there are strong connections with apoptosis, we classify hemopexin under oxidative stress as it has a high affinity with heme and reportedly plays a strong role in both heme transport and preventing heme-catalyzed oxidative damage [70]. Moreover, pyrethroids have been shown to generate free radicals and induce oxidative stress [71]. Heme is known to respond to nerve injury, and has been suggested to play a role in neurodegenerative disorders [72], and hemopexin-mediated heme transport was reported to significantly decrease levels of transferrin receptor mRNA in HeLa cells [70]. Transferrin was also identified by microarray screening as significantly upregulated by esfenvalerate exposure. The primary role of transferrin is the delivery of iron across the blood brain barrier, and its expression in brain is not only related to myelin production, but may be a permissive agent in the process of myelination [73]. Furthermore, hemopexin and transferrin reportedly act by similar receptor-mediated mechanisms [74].

#### v. Growth and development

Microarray analyses identified a gene with high homology to egg envelope glycoproteins within the zona pellucida (ZPA) referred to as choriogenins, in fish [75]. This was significantly expressed in 52-d old larvae, however, no statistical differences in expression of ZPA were measured with qPCR in 10-d old larvae exposed to esfenvalerate. Choriogenin is reportedly more sensitive to endocrine disrupting chemicals (EDCs) than estrogen receptors and vitellogenin [76]. Composed primarily of glycoproteins with various functions during fertilization and development, choriogenin has been suggested as a biomarker of exposure to endocrine disrupting chemicals, as it is induced in late stage embryos, larvae and adult male fish exposed to estrogens [76,77]. Choriogenin is synthesized in liver of adult females, in response to estrogen, transported in blood and incorporated into the fish egg envelope; chorion or zona pellucida (ZPA), an extracellular matrix that surrounds the oocyte and early embryo [78]. Expression was notably elevated at low pyrethroid concentration, and it responded in a similar fashion to creatine kinase (r = 0.93), though no significant links were identified between these two biomarkers.

#### vi. Detoxification

Pregnane × receptor (PXR), involved in the detection of toxic substances and a key regulator of xenobiotic metabolism, was identified through microarray assessments, as downregulated in 52-d old larvae. PXR is a steroid receptor and transcriptional regulator of detoxification mechanisms such as cytochrome oxidases, and phase II conjugating enzymes such as glutathione-*S*-tranferases [79,80]. Downregulation of PXR expression has been linked with growth inhibition and cell death in rats and human cell lines following exposure to medroxyprogesterone and estradiol, known PXR ligands [80], further supporting identified apoptotic responses, steroid receptor-binding and endocrine disruption activity of esfenvalerate [38,39]. PXR expression was not significantly different in q-PCR assessments of 10-d old larvae exposed to esfenvalerate, however, overall expression declined in a dose response manner, correlating with ASPA (r = 0.880; p = 0.049), making it a notable candidate of xenobiotic detection for future biomarker investigations in the delta smelt.

#### vii Osmotic Stress

A hyperosmotic glycine rich protein was identified with the microarrays as significantly downregulated by exposure to 0.0625 μg.L^-1 ^esfenvalerate in 52-d old larvae. Osmoregulation is physiologically controlled by chemical messages from the endocrine system, along with cell signalling and nerve transmission [81]. Pyrethroids have been suggested to induce osmotic imbalances in common carp larvae [82] which are linked to effects on ATPase activity responsible for maintaining the Sodium trans-membrane electrochemical gradient [83]. Larval fish are under direct exposure to osmotic stress as their endocrine system is not fully developed [84]. Parvalbumin and choriogenin expression have indicated possible effects on the endocrine system, thus expression of this hyperosmotic glycine rich protein may be directly caused by conditions affecting endocrine regulation.

#### viii Digestion

Chitinase was identified through microarray analysis as being downregulated by esfenvalerate exposure. Chitinase is the principal enzyme involved in digesting chitin, a major component of insect and crustacean exoskeleton [85]. Larval smelt were fed on artemia during the pre-exposure acclimation period. Not only chitinase but, other digestive enzymes; apolipoproteins, pancreatic enzymes, carboxypeptidase precursors and chemotrypsinogen, were also significantly upregulated following exposure in 52-d old larvae. Effects on digestion alone will undoubtedly have significant effects on growth, which when combined with hypothesized feeding reduction resulting from impaired swimming would lead to significant malnutrition. Contaminants affect a whole ecosystem, at all levels, and dramatic reductions in copepods, cladoceran and amphipod populations; organisms predated upon by the delta smelt, have been reported in the Sacramento delta [86]. Scarcity in food and reduced ingestion ability, besides digestion will significantly affect population dynamics of any specie.

## Conclusions

Microarray technology was used as an initial screening of probable genes responding to esfenvalerate exposure therefore no multiple testing correction was applied. We have, however, examined and confirmed effect of esfenvalerate upon some of the genes in a different age group of larval delta smelt, identifying significant responses that are primarily linked with swimming behavior. Some responding genes can be classified within different functional groups. Due to the measured behavioral responses, the classification approach contains a certain bias towards understanding neuromuscular effects. It is interesting that qPCR measurements have identified a greater response at the lower concentrations, implying homeostatic alterations, at environmentally relevant concentrations. Most genes did not display a desired dose response correlation associated with usable biomarkers, but did support responses within the suite of genes investigated, somewhat validating their use within a broader biomarker approach. Hemopexin for example is known to be involved in axon repair, and the myelin sheath surrounding the axon needs to be degraded for this repair to be processed, hypothesizing therefore that ASPA downregulation is resultant of neurological damage. The subsequent decrease of hemopexin expression at higher exposure concentrations, and further decrease in ASPA, may be indicative of repair impairments.

What becomes apparent from this study is that exposure to sublethal concentrations of esfenvalerate results in neurological damage and a series of compensatory molecular responses that attempt to repair nerve damage. We would hypothesize that induction of transcription of the genes encoding ASPA, hemopexin, parvalbumin and creatine kinase are part of a pathway of damage triggered repair mechanisms, responding to esfenvalerate insult. Reduction in expression of ASPA indicates that myelin sheaths may be degraded, resulting in a number of detrimental effects on the lesion sites, and similarly, muscular structure and function is being compromised as measured by alterations in titin and myozenin expression. The expression of β-microglobulins could be a compensatory reaction to toxic damage, protecting cells from infections in a susceptible immune system caused by exposure to esfenvalerate. Previous studies, carried out in esfenvalerate exposed chinook salmon have reported a compromised immunity and significantly higher susceptibility to infection [9,63]. This is particularly important in younger organisms that are generally more susceptible than adults. Furthermore, polluted waters not only contain mixtures of contaminants, but also harbor multiple pathogens that will further affect health parameters.

Behavioral endpoints, such as swimming behavior, are amongst the most sensitive and ecologically relevant parameters to assess sublethal toxicity of neurotoxic chemicals [29]. The high susceptibility of delta smelt to esfenvalerate, mediated neurological damage resulting in impaired swimming ability, also raises questions on the likely effects upon their chemosensory system; olfactory system, important in sensing reproductive pheromones, mediating reproduction. Females synthesize sex hormones stimulating male reproductive behavior [87]. Neurological damage affecting the olfactory nerves, the brain and or entire nervous system, could lead to further impairments in reproductive success following exposure to pyrethroids. Damage to the olfactory system has been used as a sublethal toxicological endpoint in fish, in studies investigating behavior following pesticide exposures [88]. Pyrethroids are known to affect the olfactory system [89]. A chemosensory gene, ictacalcin, responding to esfenvalerate exposure was also differentially expressed on the microarray. Ictacalcin is a gene originally identified in catfish (*Ictalurus punctatus*), involved in chemosensory tissues, and highly expressed in barbell, olfactory mucosa and gill [90]. Differential expression of this gene may indicate that further behavioral parameters, not investigated in this study, such as recognition, alarm response, feeding, imprinting and homing, gamete release and synchronization, contaminant avoidance [88], and other behavioral parameters that are governed by chemosensory system, could be compromised. We could speculate that outside laboratory conditions, neuromuscular and chemosensory impairments would probably result in higher ecological parameters being affected through inability to swim against water currents, making them more susceptible to predation and reducing their ability to obtain food. Furthermore, effects on chemosensory parameters would lead to migratory, reproductive, predator and contaminant avoidance impairments.

Inhibition of repair mechanisms, leading to neuromuscular damage and eventual death, was behaviorally observed throughout exposure, as impairment in swimming ability. The ability to use molecular biomarkers of neuromuscular effect further strengthens links between mechanistic effects with parameters of ecological relevance. Our study supports the use of gene expression as a productive way of understanding modes of actions of individual chemicals in endangered species. Furthermore, this screening and interrogative approach permits the identification and development of biomarkers for species of concern in which prior information is limited and allows for investigations into problems specific to the organism in question; assessing possible causes of detrimental effects and resulting influences on individual performance and hypothesizing effects upon population dynamics. A suite of biomarkers developed in this manner, though additions and subtractions are required from the presented list, could be used to aid monitor impacts of stressors upon organisms within a specific environment and could be an essential tool in determining causative factors of population decline in the delta smelt and other threatened species. The selected biomarkers clearly need to be further investigated and validated against other known contaminants, and suitability in field applications.

## Methods

### Microarray construction

We constructed a delta smelt microarray using 8448 PCR amplified fragments from a normalized cDNA library. A cDNA library was created using expressed sequence tags (ESTs) ligated into *p-BS *plasmid vectors and cloned into chemically competent *Escherichia coli *cells (BioS&T Inc, Montreal, Quebec, Canada). RNA for library construction was obtained from a number of larval, juvenile and adult delta smelt, ranging from unexposed, control conditions, to fish from exposures to high temperature (25°C), and sublethal concentrations of copper, esfenvalerate, and a six field water samples from throughout the Sacramento-San Joaquin estuary. Products were PCR amplified from 1 μl bacterial suspension, and visualized on agarose gels. Purified PCR fragments ranging in size from 1-4 kb, along with control spots, were pin-printed in duplicate onto epoxysilane coated glass slides (Schott-Nexterion, USA) in a 20 × 19 block format, with 48 blocks per microarray. Microarrays were printed using a Lucidea Array Spotter (Amersham) at the Array Core facility at UC Davis (since closed down). Microarray control spots included a number of hybridization tags comprised of a pooled PCR product from all spots on the array, *H. transpacificus *DNA, and four Spot Report System of alien PCR products from *Arabidopsis thaliana*; *CAB*, *RCA*, *RBCL *and *LPT4 *(Stratagene, USA). Blank control spots consisted of 1× Nexterion buffer solution.

### Esfenvalerate exposures

Delta smelt larvae aged 10 d and 52 d were exposed for 24 h, in two separate experiments, to a range of esfenvalerate concentrations; 0.0313, 0.0625, 0.125, 0.250 and 0.500 μg.L^-1 ^(nominal) in laboratory control water, with corresponding laboratory and solvent controls. Concentrations were measured at the start of the experiment by the Water Pollution Control Laboratory at the Department of Fish and Game (Rancho Cordova, California, USA), only single measurements were taken per treatment (results not shown), therefore we present the data in terms of nominal concentrations. Laboratory control water consisted of deionized water amended to US EPA moderately hard standards (80-100 mg.L^-1 ^CaCO_3_) and 200 μl/L methanol was used as solvent carrier. Salinity was adjusted with Instant Ocean salt to match hatchery rearing conditions (range 650 μS.cm^-1 ^to 900 μS.cm^-1^).

Average wet weights of 10-d to 52-d old larval delta smelt ranged from 0.5 to 2.5 mg respectively. Larvae were obtained from the Fish Conservation and Culture Laboratory (FCCL) UC Davis, Byron, CA, transported in cool, oxygenated 2-gallon black buckets, and held overnight in the laboratory at 17°C and a 8 h:16 h D:L light cycle. The following day, ten larvae were transferred to each 2-L beaker containing 1 L of aerated control water or esfenvalerate treatment. Each treatment consisted of 4 replicates and tests were performed at 8 h:16 h D:L cycle, at a water temperature of 17°C ± 1.2°C. The pH during the tests was 7.1 - 7.5. Dissolved oxygen levels were within the acceptable range for delta smelt (above 6.5 mg.L^-1^)[91]. Larvae were fed rotifers the day before the test start, but not during the 24 h exposure. Rotifer cultures were obtained from FCCL. During exposure, larvae were observed for aberrant swimming behavior, and surviving fish were scored after 4 h and 24 h. Swimming behavior was assessed by observing each tank for 5 min as described in Geist *et. al*. [17]. Any pronounced deviation (> 1 min) from normal (control) swimming patterns were recorded as abnormal. Effects on swimming performance (EC_50_) and mortality (LC_50_) were assessed using linear regression analysis with Environmental Toxicity Information System (CETIS) by Tidepool Scientific Software (McKinleyville, CA, USA).

Four surviving 52 d old larvae from solvent controls, and exposures to 0.0625 and 0.125 μg.L^-1 ^esfenvalerate, were used for microarray analyses, hybridized in a reference design against a pool of RNA from all treatments. Four replicate 10 d old larvae from each treatment (controls and 0.0312, 0.0625, 0.125 μg.L^-1 ^esfenvalerate) were used for biomarker analyses and gene expression verification using quantitative PCR (q-PCR).

All experiments and use of test organisms were approved by the UC Davis Institutional Animal Care and Use Committee (Animal Use Protocol for Animal Care and Use #13361). This institution is accredited by the Association for Assessment and Accreditation of Laboratory Animal Care, International (AAALAC) and has an Animal Welfare Assurance on file with the Office of Laboratory Animal Welfare (OLAW). The Assurance Number is A3433-01. The IACUC is constituted in accordance with U.S. Public Health Service (PHS) Animal Welfare Policy and includes a member of the public and a non-scientist.

### RNA isolation, cDNA synthesis and fluorescence labeling

RNA was extracted from whole, individual organisms using a standard phenol:chloroform protocol with Trizol Reagent (Invitrogen). Fifteen micrograms of total RNA were used for cDNA synthesis, spiked with control RNA (CAB, RCA, RBCL and LTP4 (SpotReport, Stratagene) and labeled with Alexa fluor dyes, using SuperScript™ Plus Indirect cDNA labeling System (Invitrogen). Each experimental sample and control was combined with a reference pool cDNA prior to hybridization using an automated Tecan HS4800 hybridization station. Slides were scanned using a GenePix 4000B scanner (Axon Instruments).

Microarray images and data from esfenvalerate exposed delta smelt can be accessed at http://www.vetmed.ucdavis.edu/apc/WernerLab/subpage/pelagic_organism_decline.html; POD archive data.

### Microarray Analyses

Normalization and analytical methods are described in Loguinov *et. al*. [32] and Smyth [92]. In brief, print tip normalization was carried out within slides and sequential single slide data analysis was carried out as an alternative to between-slide normalization. An α-outlier-generating model was used to identify differentially expressed genes by applying the following decision rule for multiple-slide data analysis: a given gene was selected as a candidate if it was detected as significantly up- or downregulated in 4 of 4 replicates (raw p-value = 0.0625 using exact binomial test and considering outcomes as Bernoulli trials). The approach did not use scale estimator for statistical inference and, therefore, it did not require between-slide normalization. This method however, detected only one significant differentially expressed candidate gene at the highest exposure concentration (0.125 μg/L^-1^), (with no significant annotation identity - see results and discussion). As a result, a second analytical method was applied to increase the number of probable genes for consideration in biomarker development. Thus we further analyzed the data using LIMMA GUI (Linear model for microarray analysis graphical user interface) [92], written in the R-programming language available through Bioconductor http://www.Bioconductor.org. Data was normalized within arrays using print-tip Lowess and between arrays applying aquantile normalization methods [92]. A linear model fit was computed using the duplicates on the arrays and the least-squares method, no multiple assessment methods were applied to eliminate false positives as our aim was to increase the number of genes available for biomarker assessment, and qualify these through quantitative PCR.

### Sequencing and Annotation

Sequencing was carried out at the CA&ES Genomic Facility, UC Davis. Basic Local Alignment Search Tool; translated nucleotide (BLASTx) searches were performed on specific fragments that responded significantly to the exposure treatments. Only genes that were differentially expressed following esfenvalerate exposure were sequenced. Sequences were annotated according to homologies to protein database searches using translated nucleotide sequences and direct nucleotide queries http://blast.ncbi.nlm.nih.gov/Blast.cgi. Sequences were only annotated if they were found to have a BLASTx match with the expect value smaller than 1 × 10^-5 ^and a score above 50.

### Functional Classifications

Differentially expressed genes were classified according to gene ontology http://www.uniprot.org/uniprot, and information gathered from literature, into functional groups. Classification was carried out based on gene expression changes in respect of control subjects, regardless of whether these were up or downregulated, or exposure concentrations. Specific genes of interest were selected for further investigation using quantitative PCR.

### Biomarker development

Genes were selected according to level of expression significance, knowledge base from literature, and functional classification. Primer and probes for qPCR analyses were designed using Roche Universal Probe Library Assay Design Center https://www.roche-applied-science.com. Designed primers were obtained from Eurofins MWG Operon http://www.eurofinsdna.com, and TaqMan probes were supplied by Roche. Sequences for all genes assessed by qPCR analyses have been submitted to genbank http://www.ncbi.nlm.nih.gov. Primers and probes, and genbank accession numbers, for investigated biomarkers are detailed in table 4. Myozenin and creatine kinase, though not resulting from the current study, were genes identified in investigations carried out during microarray development, and added to the selected biomarkers due to our interest in neuromuscular activity.

**Table 4:** Primers and probes used for molecular biomarkers: Primer pairs and TaqMan probes used in q-PCR assessments.

Accession no.	Gene		Primer Sequences	Probe No.
FJ711577	Aspartoacylase	Left:	ggaggcacacatgggaatg	109
		Right:	cttcctctgaatctctgttccattatc	
FJ711576	Parvalbumin	Left:	gaccaagacaagagtggcttca	101
		Right:	tctggcaccagcagagaagtt	
FJ711580	β-2 microglobulin	Left:	tctttcgcggtcatctttctc	22
		Right:	ggttgtggccatacacctgaa	
FJ711579	Hemopexin	Left:	catgcactacgaggacgacaag	143
		Right:	tggtagtagctgaacaccttgctg	
FJ711578	Caspase-3	Left:	gagaaccggtatgaaccaacg	159
		Right:	tccaagcttcccaaacactttc	
FJ711575	Titin a	Left:	tgatcactggcgtgaaagagg	159
		Right:	caagctcattggacagtttgagg	
FJ711581	ZPA	Left:	catgcggctgagtttggataa	106
		Right:	tgccattgatagcatcaacttca	
FJ711583	Myozenin*	Left:	ccaatgtcgtgctggtacacc	106
		Right:	ctgccagacattgatgtagcca	
FJ711584	Creatine kinase*	Left:	cgatcggcgttggagatg	163
		Right:	gccaagttcaacgagattctgg	
FJ711582	PXR	Left:	tgaggcggtggagaagag	144
		Right:	gaggcggtggagaagag	

* indicates biomarkers obtained from preliminary studies carried out during microarray development.

### Quantitative PCR

A total of 1.5 μg RNA was cDNA synthesized using random primers, and diluted to a total of 50 μl with nuclease free water to generate sufficient template for qPCR analysis. TaqMan Universal PCR Mastermix (Applied Biosystems) was used in q-PCR amplifications in a reaction containing 10mMTris-HCl (pH 8.3), 50 mM KCl, 5 mM MgCl2, 2.5 mM deoxynucleotide triphosphates, 0.625 U AmpliTaq Gold DNA polymerase per reaction, 0.25 U AmpErase UNG per reaction and 5 μL of cDNA sample in a final volume of 12 μL. The samples were placed in 384 well plates and cDNA was amplified in an automated fluorometer (ABI PRISM 7900 Sequence Detection System, Applied Biosystems). Amplification conditions were 2 min at 50°C, 10 min at 95°C, 40 cycles of 15 s at 95°C and 60 s at 60°C. Fluorescence of samples was measured every 7 s and signals were considered positive if fluorescence intensity exceeded 10 times the standard deviation of the baseline fluorescence (threshold cycle, *C*T). SDS 2.2.1 software (Applied Biosystems) was used to quantify transcription.

Quantitative PCR data was analyzed using the relative quantification 2(-Delta Delta CT) method ()([93]. In the absence of house keeping genes, expression was calculated relative to the mean of controls in respective exposures. Surviving larvae from each replicate of the 10-d old exposed delta smelt were used for q-PCR analyses. One-way ANOVA was used to assess differences in gene expression through out the exposure concentrations, and data were further assessed using Student's T-test at individual concentrations in respect to solvent controls.

## Abbreviations

EST: expressed sequence tag; q-PCR: real-time quantitative polymerase chain reaction; ASPA: aspartoacylase; ESA: endangered species act; CESA: California endangered species act; LIMMA GUI: linear model for microarray analysis, graphical user interface; BLAST: basic local alignment search tool; GABA: gamma-aminobutyric acid; NAA: *N*-acetyl-L-aspartate; ADP: adenosine diphosphate; WAP65: warm-temperature-acclimation-related-65 protein; EDC: endocrine disrupting chemical; ZPA: zona pellucida; PXR: pregnane × receptor; p-BS: p-Bluescript; IPTG: isopropyl β-galactosidase; LB: Luria Bertani; FCCL: Fish Conservation and Culture Laboratory; UNG: Uracil N-glycosylase; CT: cycle threshold.

## Authors' contributions

All authors were involved in designing the experiments. JG carried out esfenvalerate exposures recording swimming abnormality and mortality. REC designed, developed and applied the microarrays with assistance from JP and HW. REC and JP prepared, hybridized and scanned microarrays. REC and AVL performed microarray analyses. REC and LSD designed qPCR assays and performed respective assessments and analysis. REC drafted the manuscript and all authors read, contributed intellectually and approved the final manuscript.
